# Study protocol: a randomized controlled trial investigating the effects of a psychosexual training program for adolescents with autism spectrum disorder

**DOI:** 10.1186/s12888-015-0586-7

**Published:** 2015-08-28

**Authors:** Kirsten Visser, Kirstin Greaves-Lord, Nouchka T. Tick, Frank C. Verhulst, Athanasios Maras, Esther J. M. van der Vegt

**Affiliations:** Department of Child and Adolescent Psychiatry/psychology, Erasmus MC-Sophia, Wytemaweg 8, 3015 CN Rotterdam, The Netherlands; Yulius Organization for Mental Health, Dennenhout 1, 2994 GC Barendrecht, The Netherlands

## Abstract

**Background:**

Previous research shows that adolescents with autism spectrum disorder (ASD) run several risks in their psychosexual development and that these adolescents can have limited access to reliable information on puberty and sexuality, emphasizing the need for specific guidance of adolescents with ASD in their psychosexual development. Few studies have investigated the effects of psychosexual training programs for adolescents with ASD and to date no randomized controlled trials are available to study the effects of psychosexual interventions for this target group.

**Methods/Design:**

The randomized controlled trial (RCT) described in this study protocol aims to investigate the effects of the Tackling Teenage Training (TTT) program on the psychosexual development of adolescents with ASD. This parallel clinical trial, conducted in the South-West of the Netherlands, has a simple equal randomization design with an intervention and a waiting-list control condition. Two hundred adolescents and their parents participate in this study. We assess the participants in both conditions using self-report as well as parent-report questionnaires at three time points during 1 year: at baseline (T1), post-treatment (T2), and for follow-up (T3).

**Discussion:**

To our knowledge, the current study is the first that uses a randomized controlled design to study the effects of a psychosexual training program for adolescents with ASD. It has a number of methodological strengths, namely a large sample size, a wide range of functionally relevant outcome measures, the use of multiple informants, and a standardized research and intervention protocol. Also some limitations of the described study are identified, for instance not making a comparison between two treatment conditions, and no use of blinded observational measures to investigate the ecological validity of the research results.

**Trial registration:**

Dutch Trial Register NTR2860. Registered on 20 April 2011.

## Background

Adolescence is a transition phase in life with physical, emotional as well as social changes that offers several challenges for adolescents with autism spectrum disorder (ASD). People with ASD have impairments in social interaction and communication, and show limited, repetitive and stereotyped patterns of behavior, interests and activities [1]. ASD is a pervasive disorder, meaning that the impairments are usually lifelong and are expressed across multiple contexts and in multiple areas in life, although this expression can vary across the lifespan [1]. Due to the difficulties with change that many people with ASD experience, problems can increase during a transition to a new life stage, such as adolescence [2].

The physical changes in adolescence, such as the growth of secondary sex characteristics, can cause insecurity and require the learning of new routines (e.g., menstruation hygiene; [3, 4]). In addition, in adolescence the psychosexual development matures rapidly [5]: Most adolescents fall in love for the first time and have their first romantic relationships and sexual experiences, that are significant for adult functioning and for long term outcomes [6]. The psychosexual development offers specific difficulties for adolescents with ASD [7], who seem to have similar psychosexual needs to typically developing adolescents, but lack the necessary knowledge and social skills to fulfill these needs [8–10]. Therefore, adolescents with ASD usually have only few intimate and sexual experiences and can report sexual frustration and sexual preoccupations [9, 11, 12]. These problems increase the risk for adolescents with ASD to develop—or become a victim of—inappropriate sexual behavior, such as touching others inappropriately, stalking, public masturbation, sexual victimization and sexual coercion [8, 13]. Parents of adolescents with ASD frequently express concerns about the sexual behavior of their children, both about the possibility of sexual exploitation as well as concerns about the possibility their child will show inappropriate sexual behavior [14, 15].

Changes in social relations ask for new socio-communicative skills; adolescents become more independent from their parents and intimate friendships are formed [4]. These intimate friendships are important for the forming of an identity and friends are an important source of information regarding sexuality [16]. However, because adolescents with ASD generally have fewer friends and fewer close friendships than their peers without ASD [17], they receive less information about sexuality from their peers [18]. Parents report to be reluctant to provide comprehensive sexual education to adolescents with ASD [19], and therefore adolescents with ASD are more dependent on non-social sources such as the internet [20], resulting in having less (or less accurate) psychosexual knowledge compared to their peers [21].

Given the possible risks that adolescents with ASD encounter and their problems with access to reliable information resources, several studies on puberty and sexuality in adolescents with ASD emphasize the need for guidance of these adolescents in their psychosexual development [7, 14]. Interventions that focus on the transition into adolescence and in particular the psychosexual development of adolescents with ASD are however limited [8, 22]. Therefore, in order to fulfill this need, an individual training program has been developed in The Netherlands targeting the psychosexual development of adolescents with ASD: the Tackling Teenage Training (TTT) program [23, 24]. This program contains 18 one-on-one sessions, in which adolescents with ASD receive information regarding several topics (i.e., psycho-education), alternated with exercises (e.g., behavioral rehearsals, and knowledge and insight quizzes). More information on the TTT program can be found in the Intervention section. A first systematic evaluation of the effects of the TTT program on psychosexual knowledge showed that adolescents with ASD have increased knowledge of puberty and psychosexual topics after following the training program [24]. Given this promising evaluation, the effects of the TTT program are further examined within a randomized controlled design, described in this study protocol.

In the current study we investigate the effects of the TTT program on the psychosexual development of adolescents with ASD. The aims of the study were as follows: to investigate whether the TTT program (1) increases psychosexual knowledge; (2) increases skills needed for friendships and intimate relations (3) increases insight in acceptable and inappropriate sexual behaviors; (4) reduces inappropriate sexual behavior and vulnerability; (5) increases self-esteem, and (6) reduces current concerns and concerns about the future of adolescents with ASD and their parents. We hypothesized that the TTT program is not only able to reduce the difficulties of adolescents with ASD in their psychosexual development, but also to promote a positive and normative psychosexual development in adolescents with ASD.

This paper describes the study design, the participants, the content and protocol of the TTT program and the research procedures.

## Methods/Design

### Participants

Participants are 200 adolescents in the age range of 12 to 18 years with a DSM-IV diagnosis of ASD. Recruitment for the study is performed in three ways within a population of adolescents with ASD.

1. Participants are recruited among patients of Yulius (*n* = 100), a large expert mental health care institution in the South-West of The Netherlands that provides specialized care to children and adults with complex psychiatric problems.
2. Participants are recruited among students in schools with segregated settings for special education, in which students with a variety of complex psychiatric problems are educated (*n* = 50). A large number of adolescents in these settings have a diagnosis of ASD.
3. Adolescents and their parents can apply for the study through open application. Research flyers are distributed through several mental health care centers in the region among adolescents with ASD and their parents and a study website was launched (*n* = 50).

Eligibility criteria for participation are 1) a total score of 51 or above on the Social Responsiveness Scale (SRS) [25, 26], and 2) an intelligence (IQ) level in the normal to high range (total IQ > 85). More information on the SRS and the intelligence specification can be found in the Procedure section.

Written informed consent is obtained from all adolescents and their parents. This study is approved and guided by the medical ethical commission of the Erasmus Medical Center, Rotterdam (MEC-2013-040).

### Sample size

Sample size for this study was determined in advance by power calculations based on the previous pilot study results (Effect size = 0.70) [24]. In order to detect differences on the outcome measures of medium effect size between the two conditions with 80 % power (*α* = 0.05; two-sided) [27], 64 adolescents are required per condition. With an anticipated drop-out of approximately 25 % 150 adolescents will remain participating in the study, enabling us to investigate the effects of the TTT program. In addition, we will consider if it is possible to investigate the influence of potential moderators of treatment outcome.

### Intervention

The Tackling Teenage Training (TTT) program is an individual intervention with 18 sessions that cover the following topics: discussing puberty (i.e., how and with whom), appearances, first impressions, physical and emotional developments in adolescence, how to become and maintain friends, falling in love and dating, sexuality and sex (e.g., sexual orientation, masturbation, and intercourse), pregnancy, setting and respecting boundaries and safe internet use [23, 24, 28]. An overview of the sessions is displayed in Table 1. The TTT program is developed for adolescents with ASD from 12 and 18 years old with a normal or high intelligence. During the TTT program, the trainer and adolescent discuss one session per week, in which one topic is discussed and exercises are practiced in a structured manner in approximately 45 min. The training program can be provided in the school setting, in outpatient or inpatient facilities or at the home of the adolescent (if certain conditions can be met, such as a confined room and no disturbance from family members). All sessions are structured in the following way: First, the take-home assignment of the last session is discussed. After this, the adolescent receives information regarding different subtopics of the session (i.e., psycho-education), alternated with exercises (e.g., behavioral rehearsals, and knowledge and insight quizzes). At the end of each session, the adolescent receives a take-home assignment in which the topic of that session needs to be discussed or practiced outside of the context of the training, for instance conducting a small interview with one of the parents on the topic of the session, or arranging a get-together with a friend.

**Table 1:** Overview of sessions and themes in the Tackling Teenage Training program

Sessions	Themes
Session 1 Talking about puberty	Discussing puberty
Session 2 This is me	Appearances
Session 3 A good first impression	First impressions
Session 4 What do you call that?	Naming body parts
Session 5 Changes during puberty in boys	Male physical changes
Session 6 Changes during puberty in girls	Female physical changes
Session 7 Making love to yourself	Masturbation, rules and hygiene
Session 8 Friendship	How to become and maintain friends
Session 9 Being in love and stuff…	Falling in love
Session 10 Doubts and confusion during puberty	Sexual orientation
Session 11 Being in love and dating	Falling in love and dating
Session 12 Safe sex	Sexual intercourse, contraception and STD
Session 13 The first time	Sexual intercourse
Session 14 Pregnancy and birth	Pregnancy
Session 15 Where do you draw the line?	Setting and respecting boundaries
Session 16 Yours and other people’s boundaries	Setting and respecting boundaries
Session 17 Internet and making contact	Safe internet use
Session 18 Bad boyfriends (session for girls)	Abusive boyfriends

The training starts and ends with an appointment with the adolescent and the parents, who are weekly informed by mail on the progress of the training by the trainer, via structured contact reports. The contact reports consist of information on the topic of the session (i.e., psycho-education) and the take-home assignment of the child, in order to inform parents, but also to stimulate rehearsal of the skills that were taught in the session within the home environment. In addition, particularities (i.e., the strengths and difficulties of the child in the current session) are communicated with parents. The contact reports allow parents to be prepared for any questions or remarks of the adolescent in between sessions and foster an environment in which the adolescent can discuss or practice topics of the TTT program within the home environment and/or broader social environment with guidance of parents to enhance generalization to other contexts outside the setting of the training.

All trainers that provide the TTT program in the study have a Bachelor or Master in psychology or social services and experience in working with people with ASD. They have taken the 2 day train-the-trainer course, in which they received information on the psychosexual development in general, possible difficulties in the psychosexual development for adolescents with ASD and they were taught how to provide the training program in a standardized way, with particular suggestions on how it can be customized to the individual needs of the adolescent if necessary. In addition, all trainers participate in interdisciplinary meetings every 3 months, in which questions and case reports can be discussed with other trainers and the developers of the training program. The protocolized manual of the TTT program [23] is available in Dutch, English, Greek and Spanish.

For each training session*,* information about the motivation, resistance and openness of the adolescent, and the difficulty of the material for the adolescent, according to the trainer, is registered by the trainer. Motivation of the adolescent is scored by the trainer on a scale from 0 to 10 (i.e., 0 = no motivation to 10 = very motivated). The trainer rates how much resistance the adolescent shows and how much information the adolescent shares during the sessions, again on a scale from 0 to 10 (i.e., 0 = no resistance at all to 10 = very much resistance / 0 = very guarded to 10 = very open). Last, the trainer rates how difficult that particular session is for the adolescent on a scale from 0 to 10 (i.e., 0 = very easy to 10 = extremely difficult). All session scores are subsequently summed and then averaged to compute an index of overall motivation, resistance, difficulty and openness. During the training, neither the parents nor the adolescent are informed about these scores.

Furthermore, after each session the trainer reports to what extend the TTT protocol was followed through standardized short evaluation forms, in order to register adjustments that are made to the TTT protocol. Examples of adjustments are: changing the order of the sessions, spending more time to discuss a theme, or leaving out (additional) texts and questions. In this way, the adherence to the TTT protocol by the trainers can be monitored for each participant and each session.

After ending the training, the parents and adolescent fill out an evaluation form with 16 questions, for a more subjective evaluation of the topics the adolescent learned about in the TTT program and how parents and adolescents rate and appreciate the training (e.g., the texts, the illustrations, the relationship with the trainer, the communication with the trainer, the take-home assignments) on a scale from 0 to 10.

### Procedures

**Fig. 1:**
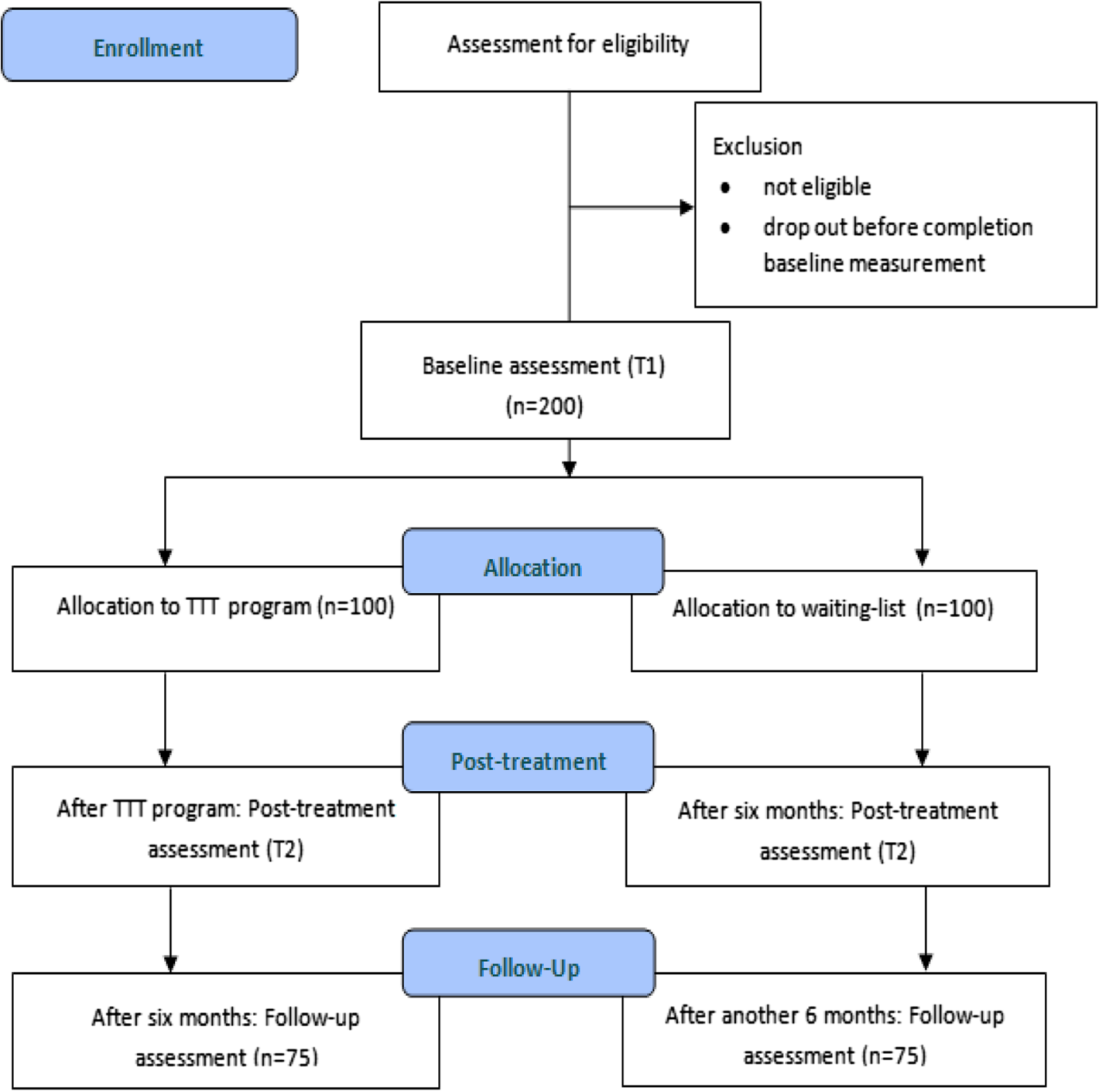
CONSORT Study Flow Chart for the Tackling Teenage Research

Figure 1 shows the planned CONSORT (Consolidated Standards of Reporting Trials) Study Flow Chart for this study. After application, eligibility criteria are confirmed. For this, all parents fill out the Social Responsiveness Scale (SRS) [25, 26], together with the consent forms. The SRS is a 65-item questionnaire that covers the domains of social behavior, language, and repetitive/stereotypic behavior that are characteristic of ASD. A total score and five treatment subscales can be derived from the SRS: Social Awareness, Social Cognition, Social Communication, Social Motivation, and Autistic Mannerisms. The SRS provides a dimensional measure of autistic traits, with higher scores on the SRS reflecting greater degree of social impairment. Internal consistency of the SRS is excellent (α = 0.97) [25]. We use a total score of 51 or above on the SRS, because this is the preferred cut-off point based on research among clinical referrals and children from the Dutch general population [26].

Total IQ, verbal IQ (VIQ) and performance IQ (PIQ) are taken from the medical file of the adolescent and are used if the assessment is not older than two years old and if a valid and reliable instrument was used (for instance one of the Wechsler intelligence scales). In cases where no or no recent IQ measurement is available, IQ is assessed using the Wechsler Abbreviated Scale of Intelligence (WASI) [29]. The WASI consists of four subtests: Vocabulary, Block Design, Similarities, and Matrix Reasoning. These four subtests compose a total IQ, a VIQ and a PIQ. The internal consistency, test-retest and inter-rater reliability of the WASI are good and it correlates strongly with full-scale Wechsler Adult Intelligence Scale-III (WAIS-III) IQ scores (α = 0.87) [29].

In the current study we use multi informant questionnaires, both parent-report and self-report. The use of self-report in research with people with ASD is sometimes debated, because of their problems with identifying and describing feelings and emotions of others and themselves [30]. Still, self-report gives an indication of the perception of behavior and feelings of people with ASD [14], which is indispensable when studying intimate and personal themes, such as pubertal development and psychosexual functioning. From research it also becomes clear that although people with ASD tend to have difficulty identifying, verbalizing, and analyzing their emotions, they experience emotions just like everyone else and that their self-reports have good internal reliability [30–32].

All participating adolescents and their parents fill out questionnaires at three time points during 1 year: at baseline (T1), after inclusion criteria are confirmed; post-treatment (T2), directly after the TTT program (for adolescents in the intervention condition), or after 6 months on the waiting-list (for adolescents in the control condition), and for follow-up (T3), 6 months after the TTT program (for adolescents in the intervention condition), or after 12 months on the waiting-list (for adolescents in the control condition). The adolescents fill out the questionnaires in the company of a research assistant (who is available for questions of the adolescent). The research assistants are blind to the condition of the adolescents. Parents fill out the questionnaires via the internet.

### Randomization

Simple equal randomization (1:1) takes place at an individual level, after the baseline measurement (T1). Adolescents are randomly assigned to the intervention condition (*n* = 100) or the control condition (*n* = 100). A computerized allocation scheme is used, authorized by an investigator with no involvement in the current research. Adolescents in the intervention condition start the TTT program shortly after the randomization. The adolescents in the control condition are placed on a waiting-list. During the study, adolescents in both conditions can continue other interventions and medications.

### Outcome measures

In order to investigate the effect of the TTT program on the psychosexual development of the adolescents with ASD, we used the Teen Transition Inventory (TTI) (Dekker, van der Vegt, van der Ende, Tick, Louwerse, Maras, Verhulst, Greaves-Lord. The development and testing of the Teen Transition Inventory: A self- and parent-reported assessment of psychosexual functioning in adolescents with autism spectrum disorder and typically developing peers, in preparation). Table 2 provides an overview of all questionnaires and tasks that we use in the study. The TTI is a self-report (186 items) and parent-report (148 items) questionnaire, that covers the experience of several transitions in adolescence, such as the school transition, physical change, psychosexual functioning and psychosexual problems and worries of the adolescents and their parents. The psychosexual functioning part of the TTI contains nine scales and several separate items that represent the three domains of psychosexual functioning: sexual selfhood, sexual socialization, and sexual behavior [5, 7] (see Table 3 for an overview). All items are scored on a 3-point scale (0 = not true, 1 = somewhat or sometimes true, 2 = very true or often true), with the exception of a minority of separate items (i.e., age of onset). The scale scores reflect the average score of all the items on the scale, with scores ranging between 1 and 3. The scales showed sufficient to good internal consistency (Dekker et al, in preparation). The self-report and parent-report version of the TTI is administrated at T1, T2 and T3.

**Table 2:** Overview of measures in the Tackling Teenage Research

Measures	For adolescents	For parents
Inclusion	WASI^a^	SRS
T1	ADOS-2	TTI parent-report
	Psychosexual knowledge test	CBCL
	TTI self-report	Child & family characteristics
	Flag system	
	NPV-2—self esteem scale	
T2	Psychosexual knowledge test	TTI parent-report
	TTI self-report	SRS
	Flag system	CBCL
	NPV-2—self esteem scale	
T3	Psychosexual knowledge test	TTI parent-report
	TTI self-report	SRS
	Flag system	CBCL
	NPV-2—self esteem scale	

*WASI* Wechsler Abbreviated Scale of Intelligence, *SRS* Social Responsiveness Scale, *ADOS-2* Autism Diagnostic Observation Schedule-2, *TTI* Teen Transition Inventory, *CBCL*  Child Behavior Checklist

^a^Only administered when no recent IQ measurement is available

**Table 3:** Psychosexual functioning part of the Teen Transition Inventory

Domain	Scale/items	Parent-report	Self-report
Sexual socialization	Friendship skills of child	5 items	5 items
	Social acceptance by peers	3 items	6 items
	Romantic ability of child	-	3 items
	Openness about intimacy	4 items	3 items
	Adequate dealing with boundaries	8 items	-
Sexual selfhood	Bodily perception	3 items	6 items
	Self-esteem	-	12 items
	Perceived social competence	-	12 items
	Sexual knowledge of the child according to parent	9 items	-
Sexual behavior	Separate items on age or context appropriate behaviors, intimate experiences including age of onset and sexual orientation	33 items	41 items

#### Psychosexual knowledge

To investigate whether the TTT program improves knowledge regarding psychosexual themes, a psychosexual knowledge test for adolescents [24] is administered at T1, T2, and T3. The psychosexual knowledge test consists of 35 multiple choice questions and 2 open-ended questions in which parts of the (male and female) genitals have to be named correctly. Each question is rated as being either correct or incorrect, which leads to a score of one point per question. The total score on the psychosexual knowledge test represents the total number of correct answers, ranging from 0 to 37.

Parents are questioned about the knowledge of their children in the ‘Sexual knowledge of the child according to the parent’ scale of the TTI (Dekker et al, in preparation).

#### Skills needed for friendships and intimate relations

The ‘Friendship skills’ scale (self-report and parent-report version) of the TTI is used to measure the ability of the adolescents to make and maintain friendships. The ‘Romantic ability’ scale (self-report version) of the TTI is used to measure the self-perceived romantic relational skills of the adolescents (Dekker et al, in preparation).

The Social Responsiveness Scale (SRS) [25] is used as a measure of ASD symptom severity and social impairments [33], but the treatment subscales can be used for assessment of treatment effect. The four treatment subscales in the social domain determine to what extent an adolescent is aware of social cues in his environment (Social Awareness), can appropriately interprets those cues (Social Cognition), is capable of a reciprocal reaction (Social Communication), and is motivated to engage in social-interpersonal behavior (Social Motivation) [34]. The treatment subscales of the social domain of the SRS have been shown to be sensitive to changes in social functioning among children with ASD [35–37] and therefore we administer the SRS also at T2 and T3.

#### Insight in acceptable and inappropriate sexual behaviors

To investigate whether the TTT program increases insight in different sexual behaviors, the ‘Adequate boundaries’ scale (parent-report version) (Dekker et al, in preparation) is used. In addition to this, the flag system (Dutch: het Vlaggensysteem) [38], is administrated at T1, T2 and T3 to measure the accuracy of judgment of acceptable and inappropriate sexual behavior. The flag system contains drawn illustrations with children and adolescents displaying sexual behavior in different situations and is developed to discuss a range of sexual behaviors with children or adolescents. The scoring of the flag system is based on six criteria for judging sexual behavior: consent, equality, coercion, appropriate for age, appropriate for context, and self-respect [38, 39]. The developers of the flag system used these six criteria to define four flags (green, yellow, red, and black). An expert panel of child health care professionals chose the ‘correct’ or most accurate flag for each illustration during the 2 year development of the flag system. In the current study the flag system is registered individually. The research assistant first explains the meaning and use of the four flags and then shows the adolescent 23 illustrations one-by-one. With each illustration, the research assistant asks the adolescent to choose a flag, best judging the sexual behavior shown in the illustration. The research assistant writes down the chosen flag, without giving feedback to the adolescent during the test, and continues to the next illustration. The chosen flags of the adolescents are compared to the chosen flags of the expert panel, and scored as either correct or incorrect. An incorrect flag could indicate a milder judgment or a stricter judgment, compared to the normative judgment of the expert panel. We calculate the average percentages of accurate judgments, milder judgments and stricter judgments for each illustration of the flag system.

#### Inappropriate sexual behavior and vulnerability

To investigate whether the TTT program reduces inappropriate sexual behavior and vulnerability, several separate items of the TTI (self-report *n* = 8 and parent-report version *n* = 12) (Dekker et al, in preparation) are used. Example items from the parent-report TTI are: “My child touches others where they do not like to be touched”, “My child is able to set his/her boundaries regarding intimate contact”, “Does your child ever masturbate at inappropriate times, in inappropriate ways or places?”. Some example items from the self-report TTI are: “I keep contacting someone, even though that person has indicated he/she does not want any contact with me”, “I touch others in places where they do not want to be touched”, and “In general I am good at letting people know what I am comfortable with and what I am not comfortable with”.

Furthermore, we use the Sex Problems scale of the Child Behavior Checklist (CBCL) [40, 41] at T1, T2 and T3, to determine the occurrence of psychosexual problems. The Sex Problems scale consists of five items regarding sexual problems, thinking of sex too much, playing with own genitals in public, playing with own genitals too much and the desire to be from the other gender [40, 42]. Parents rate each item on the CBCL on a 3-point scale (0 = not true, 1 = somewhat or sometimes true, 2 = very true or often true). Research that included the CBCL Sex Problems scale for the purpose of measuring psychosexual problems by means of parent report has supported the content & concurrent validity of this scale as well as the discriminant validity [42, 43].

#### Self-esteem

To investigate whether the TTT program increases the adolescents’ self-esteem the Self-esteem scale of the Dutch Personality Questionnaire (NPV-2) [44, 45] is administrated at T1, T2 and T3. The NPV-2 consists of 132 items, scored on a three-point scale (true, not true and not false, or false) and measures the following 7 personality traits: inadequacy, social anxiety, rigidity, resentment, selfishness (complacency), dominance, and self-esteem. In the current study, we use the self-esteem scale, consisting of 19 items that the adolescents answer about themselves. Examples of questions are: “I often feel insecure”, “I can handle jokes” and “I can accomplish what I want”. Norm data of multiple populations (e.g., general and psychiatric patients) are available and reliability and validity of the self esteem scale are good (α = 0.86) [45].

In addition, the TTI scales ‘Perceived social competence’ (self-report version) and ‘Self-esteem’ (self-report version) are used to measure changes in the self-esteem of the adolescents (Dekker et al, in preparation).

#### Concerns of the adolescents with ASD and their parents

To investigate whether the TTT program can reduce current concerns and concerns about the future for adolescents with ASD and their parents, we use several questions in the TTI (parent-report version *n* = 8; self-report version *n* = 7) about current and future concerns, for instance: “I worry about the vulnerability of my child regarding social relationships in general”, “I worry about my child on the internet”, “I worry if my child will live with a partner” and “I believe it is possible that I will have social relationships in the future” (Dekker et al, in preparation).

### Other measures

We administer additional measures to investigate the influence of (1) ASD severity, (2) emotional and behavioral problems, and (3) child and family characteristics on the effectiveness of the Tackling Teenage Training program.

#### ASD severity

The Autism Diagnostic Observation Schedule-2 (ADOS-2) [46, 47] is administered at the baseline measurement (T1). The ADOS-2 is a semi structured, standardized measure to assess social interaction, communication, play, and stereotyped behavior for individuals suspected of having ASD. The ADOS-2 has four modules, each designed to be administered to different individuals according to their level of expressive language. Psychometric properties of the ADOS-2 are good [46]. In this study the ASD severity is registered using the calibrated severity scores [48]. The ADOS-2 is administered by psychologists who have completed the 2-day research-training and have achieved sufficient reliability for administration and coding.

#### Emotional and behavioral problems

We expect that emotional and behavioral problems of the adolescents might influence the effects of the Tackling Teenage Training program. Therefore, we use the Child Behavior Checklist (CBCL) [40, 41] at T1 to register emotional and behavioral problems of the adolescents. The CBCL assesses anxious/depressed problems, withdrawn/depressed problems, somatic complaints, social problems, thought problems, attention problems, aggressive and delinquent behavior problems. These scales are combined into an internalizing score (anxious/depressed problems, withdraw problems and somatic complaints), externalizing score (aggressive and delinquent behavior problems), and total problems score. Psychometric properties of the CBCL are good [40, 41].

#### Child and family characteristics

Demographic characteristics, such as age, gender, family situation are administered in the baseline measurement (T1).

Furthermore, socio-economic status (SES) is determined by income level, educational level of both the mother and the father (or other caregivers), and occupational level of each primary caregiver, using the International Standard Classification for Occupations [49].

Ethnicity is determined by the birth country of both parents. If both parents are born in The Netherlands, the adolescent is categorized as ethnicity ‘Dutch’. If one or both parents are born in another country than the Netherlands, the adolescent is categorized as ethnicity ‘non-Dutch’.

In order to determine previous sexual education of the adolescent, the adolescents are asked if they previously received education or training on the subject of friendship, intimacy or sexuality, and if yes, from who they received this education or training (e.g., from parents, friends, in school, the internet). Parents are also asked if their child previously received education or training on the subject of friendship, intimacy or sexuality and if yes, from whom their child received this education or training.

Family values and attitudes regarding sexuality are determined with the TTI, with the ‘Openness about intimacy’ scale. Example items in this scale are: “Within our family we discuss sexuality”, “My child takes the initiative to talk about sexuality with me/us” and “I discuss my feelings and/or questions about intimacy/sexuality with my parents” (self-report and parent version) (Dekker et al, in preparation).

### Data analyses

Differences in baseline characteristics (age, gender, total IQ, ADOS severity score, scores on the SRS at T1 and the psychosexual knowledge test at T1) are examined with independent samples t-tests or chi-square tests. Baseline descriptors that differ significantly are taken into account in following analyses.

To investigate the effectiveness of the TTT program, we explore both within-subject effects (changes within adolescents in both conditions) and between-subject effects (changes between adolescents in the control condition versus the intervention condition). ‘Time’ is the within-subject variable, with three levels: T1 (baseline), T2 (effect), and T3 (follow-up). ‘Condition’ is the between-subject variable, with two levels: intervention condition and control condition. We use several continuous outcome measures: psychosexual knowledge, the scales of the Teen Transition Inventory (TTI), insight in acceptable and inappropriate sexual behaviors, self-esteem, and concerns about the future. And we use several dichotomous outcome measures, such as the separate items of the TTI measuring inappropriate sexual behavior. We correct for multiple testing using Bonferroni correction. Statistical analyses are performed using SPSS 19.0 statistical software (SPSS) [50] and are two-sided, with a level of significance of *α* = 0.05.

In addition to this, we investigate if potential predictors of treatment outcome (ASD severity, emotional and behavioral problems, and child and family characteristics) influence the effects of the TTT program.

### Drop out analyses

For participants in both conditions (the intervention condition and the control condition), we examine the differences between the drop outs and the full participating individuals concerning several descriptors (age, gender, total IQ, ASD symptom severity) to investigate whether drop-out is selective or random.

## Discussion

The study described in this study protocol is the first randomized controlled trial (RCT) to investigate the effects of a psychosexual training program, the Tackling Teenage Training (TTT) program, for adolescents with autism spectrum disorder (ASD). The current RCT has a number of methodological strengths: The conduction of a first systematic evaluation of the outcome of the TTT program on psychosexual knowledge [23] in the years before the RCT contributed to the quality and the practicability of the TTT protocol, and made it possible to optimize the procedures and measures in the research protocol. The used sample size in the RCT will give the study enough power to investigate the effects of the TTT program and the wide range of functionally relevant outcome measures will guarantee the clinical importance of the results. In addition, we use multiple informants (self-report and parent-report questionnaires). As a final strength of the study, the adherence to the tested training protocol is carefully monitored. However, we also acknowledge certain limitations of the current study: Because in the Netherlands no other protocolized psychosexual training program is available, it is not possible to make a comparison between two interventions, and therefore the control condition is a waiting-list condition. Furthermore, because validated blinded observational measures to investigate changes in psychosexual functioning and sexual behavior are currently not available, we are limited to using parent- and self-report questionnaires reporting on psychosexual functioning and sexual behavior of the adolescents.

In conclusion, the results of the study described in this study protocol will be a valuable contribution to the growing knowledge of the psychosexual development of people with ASD, and aims to ultimately improve the support adolescents with ASD receive during the challenging life phase of adolescence.

### Trial status

Patient recruitment for this study lasted from January 2012 to April 2014 and 210 adolescents and their parents gave permission to participate and were assessed for eligibility. From these, 188 adolescents met the inclusion criteria, completed the baseline measurement (T1), and were randomized. The effect measurement (T2) was completed in November 2014 with data from 168 adolescents and the follow-up measurement (T3) was completed in June 2015 with data from 160 adolescents. All data is currently analyzed.

## Abbreviations

ADOS-G: Autism Diagnostic Observation Schedule–Generic
ASD: Autism spectrum disorder
CBCL: Child Behavior Checklist
HRQOL: Health related quality of life
SES: Socio-economic status
SRS: Social Responsiveness Scale
RCT: Randomized controlled trial
TTI: Teen Transition Inventory
TTT: Tackling Teenage Training
